# Development of a Multilingual Web-Based Food Frequency Questionnaire for Adults in Switzerland

**DOI:** 10.3390/nu15204359

**Published:** 2023-10-13

**Authors:** Sarah T. Pannen, Roland Gassmann, Robert Vorburger, Sabine Rohrmann, Janice Sych, Nina Steinemann

**Affiliations:** 1 Division of Chronic Disease Epidemiology, Epidemiology, Biostatistics and Prevention Institute, University of Zurich, Hirschengraben 84, CH-8001 Zurich, Switzerland; sarahtheresa.pannen@uzh.ch (S.T.P.); nina.steinemann@uzh.ch (N.S.); 2 Institute of Computational Life Sciences, ZHAW School of Life Sciences and Facility Management, Schloss 1, CH-8820 Wädenswil, Switzerland; garo@zhaw.ch (R.G.); voru@zhaw.ch (R.V.); 3 Institute of Food and Beverage Innovation, ZHAW School of Life Sciences and Facility Management, Grüentalstrasse 14, CH-8820 Wädenswil, Switzerland; janice.sych@zhaw.ch

**Keywords:** dietary assessment, diet, FFQ, web-based, nutritional epidemiology, development, usability

## Abstract

Dietary assessment is a major challenge in epidemiological research and is associated with a high time and financial burden. Automated food frequency questionnaires (FFQs) have the potential to rapidly collect dietary intake data in large studies while reducing human error risk during data processing. We developed a semiquantitative, multilingual, electronic FFQ for real-time dietary intake assessment in the Swiss adult population, called “Swiss eFFQ”. The iterative development process involved stages of content identification, construction, pretesting, translation, and adaptation of the FFQ. Using 24 h dietary recalls from 2085 participants aged 18–75 years from a nationally representative survey, we conducted a stepwise regression analysis to identify foods contributing to >90% of the variance in intakes of energy and six nutrients. All 118 foods identified in the overall cohort or in any of the Swiss linguistic regions were selected and standardized to define the comprehensive 83-item food list, covering >90% of the intake of key nutrients in the entire study population. Once validated, the Swiss eFFQ can be used to classify individuals based on their habitual diets. The methodology described in this paper enhances the transparency of the Swiss eFFQ and may help researchers to develop multilingual dietary assessment tools for other populations.

## 1. Introduction

Diet is an important modifiable lifestyle risk factor playing a key role in the development and progression of noncommunicable diseases worldwide [1]. However, valid estimation of dietary intake is one of the major challenges in dietary research [2,3]. Despite the known risks of systematic and random error associated with food frequency questionnaires (FFQs), they have long been the tool of choice for investigating the effects of diet on health and disease in epidemiological research [4]. The principle of FFQs is a predefined list of foods presented to the respondent who then indicates the usual frequency of consumption of each food over a specified time period. This information is then utilized to estimate the individual’s habitual daily dietary intake by using food composition databases [5]. Web-based FFQs offer advantages such as the possibility of self-administration by a wide range of individuals, high user friendliness, low respondent burden, semi-automated cost- and time-efficient data collection, the ability to control for incomplete data entry, and the inclusion of estimation aids for respondents [6,7]. Given that eating habits and dietary trends are culture-specific and change over time, FFQs should be adapted, updated, and validated for the specific target population [5,8,9]. In culturally diverse populations such as Switzerland, this is a particular challenge [10].

Recently, there has been a growing interest in understanding the dietary habits of the Swiss population, driven by research highlighting the potential health benefits and risks associated with certain foods and dietary patterns [11,12,13,14]. For example, a recent study conducted in the French-speaking part of Switzerland suggested that plant-based diets are associated with a better cardiovascular risk profile [15], and investigations of adherence to certain dietary patterns, such as the Mediterranean diet or the Alternate Healthy Eating Index, have supported the hypothesis that dietary patterns affect chronic disease mortality in Switzerland [12,14,16]. Meanwhile, concerns have been raised about the potential detrimental effects of ultra-processed food consumption and its association with excess body weight observed in Swiss women [17]. Moreover, adverse health effects related to alcohol consumption and cancer mortality were reported [18]. The above results emphasize the need among researchers and policy makers to better understand food consumption patterns, enabling the development of targeted public health interventions to promote healthier dietary choices.

Analyses of sociodemographic determinants of diet based on data from the first Swiss National Nutrition Survey *menuCH* found major differences in diet and diet quality scores among the three linguistic regions of Switzerland [19,20,21]. For example, Chatelan et al. observed regional differences in the consumption of 18 out of 31 food subgroups [20]. Furthermore, they reported that the diets of Swiss residents living in the French- and Italian-speaking regions appeared to adhere more to a Mediterranean diet (lower in milk and higher in vegetable oil, fish, poultry) compared to those living in the German-speaking region [20]. Interestingly, it has been suggested by the authors that the observed differences in the consumption of certain foods and dietary patterns may be culturally influenced by the respective neighboring countries, namely Germany, France, and Italy [20,21]. A previous study that compared risk factors and mortality characteristics in the German- and French-speaking parts of Switzerland observed mostly similar patterns between Germany and France, respectively. This highlights the potential of Switzerland to study the role of culture-related risk factors while eliminating bias from differences in national health policies [13]. Therefore, Switzerland, with its culturally diverse dietary habits and significant regional differences in chronic disease profiles, represents an ideal setting for epidemiological research on associations between diet and chronic diseases [13,22]. As differences in dietary habits affect food and nutrient intake and, thus, individual risk profiles, flexible assessment and monitoring of dietary intake is crucial. Digital FFQs have the potential for the semi-automated collection and processing of dietary data [6]. However, the only FFQs available in Switzerland are paper-based or outdated, and a digital tool with a solid nutritional basis that can be used in nationwide studies is currently lacking [23,24].

Therefore, our aim was the data-driven development of a web-based, self-administered FFQ that takes into account culturally specific foods and is optimized for the dietary assessment of adults living in Switzerland. The electronic FFQ, called “Swiss eFFQ”, will promote and strengthen epidemiologic research on the role of diet in health and chronic disease risk.

## 2. Materials and Methods

### 2.1. Data-Driven Development of the Swiss eFFQ

**Figure 1:**
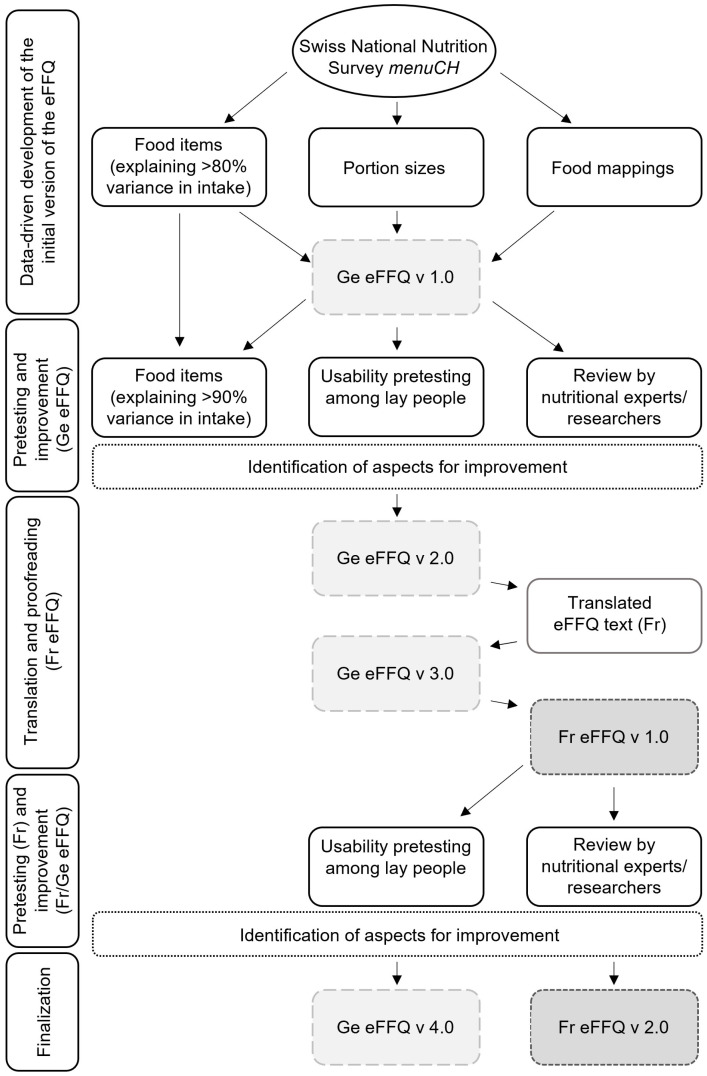
Overview of the steps in the development of the multilingual Swiss eFFQ. eFFQ: electronic food frequency questionnaire; Fr: French; Ge: German; v: version.

The development process of the Swiss eFFQ involved several stages, including iterative phases of content identification, review, pretesting, and adaptation of the Swiss eFFQ. A summary of the major development steps is shown in Figure 1.

#### 2.1.1. Data Source

A data-driven approach using data from the first Swiss National Nutrition Survey *menuCH* was used to systematically develop the Swiss eFFQ [20,25]. This cross-sectional, population-based survey was conducted between January 2014 and February 2015 in a representative sample of Swiss adults. Dietary intake was assessed by trained dietitians using two 24 h dietary recalls (24 h-DR): the first was conducted in person and the second by telephone, with an interval between the two non-consecutive assessments ranging from two to six weeks. Recall data were coded using the internationally validated GloboDiet^®^ software (v CH-2016.4.10, developed by the International Agency for Research on Cancer (IARC) in Lyon, France; former software name: EPIC-Soft^®^ [26]), optimized for Switzerland (GloboDiet^®^ trilingual databases dated on 12 December 2016) in collaboration with IARC (Lyon, France) and the Federal Food Safety and Veterinary Office (FSVO, Bern, Switzerland). To facilitate the quantification of consumed amounts, participants had access to a food picture book with 119 series of five to six portion-size images and approximately 60 actual household measurements [27]. Details of the study design and main results have been published elsewhere [19,20]. In brief, a stratified random sample (35 strata: 7 major areas of Switzerland × 5 age categories) of 2085 participants from the three linguistic regions (German-, French-, and Italian-speaking parts) of Switzerland, aged 18–75 years, was included in the study. Data from all study participants were used in the present study: complete dietary intake data (2 × 24 h-DR) were available for 2057 study participants and only one assessment was available for 28 participants. Due to missing micronutrient data in the food composition database used to estimate the nutrient contents of the 24 h-DR, missing micronutrient values for each food item were imputed by using the median micronutrient value from the corresponding food categories according to a previously used procedure [19]. The *menuCH* survey was conducted according to the Declaration of Helsinki, and all procedures were approved by the ethics committee of the Canton of Lausanne (lead committee, Protocol 26/13) and by the corresponding regional ethics committees. All analyses were performed with R software (version 4.0.2 for Windows, R Foundation for Statistical Computing, Vienna, Austria).

#### 2.1.2. Data-Driven Content Identification

Individual foods, beverages, and mixed dishes reported during the 24 h-DR of the entire *menuCH* study population were used as the basis for the Swiss eFFQ food item selection procedure. Prior to analysis, these food codes were manually standardized and grouped based on nutritional similarities.

To define the food items for the Swiss eFFQ food list, forward stepwise regression was used to identify the foods that explained most of the variance in intakes of energy and six key nutrients (carbohydrates, fat, protein, fiber, saturated fatty acids, and vitamin C) [5,9]. An analysis was conducted in the entire cohort and stratified by linguistic region. Food items reported less than ten times (total number of observations < 10) were excluded from the analysis. Foods that cumulatively contributed to >90% (adjusted R^2^ > 0.9) of the variance in intake of energy or any of the key nutrients for the entire cohort or any of the linguistic regions were selected for inclusion in the Swiss eFFQ food list. Aiming for a medium-length FFQ, the length of the food list was then shortened by combining foods into appropriate food items (e.g., different types of cake were combined into a “cake” food item).

Since the data-driven selection of foods for the Swiss eFFQ was based solely on their contribution to variance in energy and nutrient intakes, additional a posteriori analyses were conducted to examine the contribution of all food items included in the Swiss eFFQ to absolute intakes of energy and nutrients (carbohydrates, fat, protein, fiber, saturated fatty acids, and vitamin C) in the population (population-based approach) [8].

For each food item, the population-based composition of sub-foods that cumulatively contributed to >80% of the consumption frequency of the food item in the entire population was calculated. These percentages, hereafter referred to as “food mappings”, are used for the automatic calculation of daily nutrient intakes in the Swiss eFFQ (see Section 2.1.5). Each food mapping component (sub-food) was matched to the corresponding food from the food composition database most frequently used to calculate the nutrient intake of that food in the *menuCH* survey. The data-driven matched list was reviewed by the responsible nutritionists (S.P., N.S.), and adjustments were made as needed.

In the Swiss eFFQ, one standard portion size per food item is used. To estimate the population-specific food-portion sizes, food intakes reported in *menuCH* were summed by eating occasion, and the median intake (weighted for age group, sex, major region, marital status, nationality, and household size) of the entire population was calculated for each FFQ food item [28]. The values were compared with standard portion sizes of the Swiss population and cross-checked for plausibility by the responsible nutritionists (S.P., N.S.) [29].

#### 2.1.3. Structure of the Swiss eFFQ and Its Questions

The Swiss eFFQ collects food intake data over the past four weeks using ten (10) frequency options, ranging from never to more than five times per day (Figure S1). For some foods for which a higher level of detail is of interest, additional information is collected on the specific food variants usually consumed by asking respondents to indicate the most commonly consumed food variants, using two different question formats: (Type 1) to differentiate between two food variants (e.g., sugar-free vs. sugar-containing sweetened beverages), the respondent is asked to indicate the relative frequency of consumption of variant A vs. variant B by selecting one of five frequency categories (never, rarely, sometimes, often, and always); and (type 2) to choose from more than two food variants (e.g., milk/milk alternatives), the respondent is asked to select all of the food variants he or she consumes from a list of options. A food image is graphically displayed next to each food frequency question, depicting an example food at the standard serving size. The photos were selected from the *menuCH* Swiss photo book (~75%) and from the previous FFQ for German-speaking Swiss adults (~25%) [23,27].

In addition to the food frequencies assessed at the food item level, the Swiss eFFQ also assesses the intake frequency of some food categories which tend to be over- or under-reported (e.g., vegetables) or are consumed in high daily frequency (e.g., water). These correction questions ask respondents to report the timeframe (month, week, or day) and frequency of consumption (1–10) of the item.

#### 2.1.4. Technical Aspects in the Development of the Swiss eFFQ

The open-source content management system (CMS) and PHP framework Drupal (v 9.5.0) was used to set up the web platform of the Swiss eFFQ. PHP (v 8.1.24) was used for the server-side programming (e.g., calculation of daily food intake), the configuration of the platform (e.g., structure of the questionnaire) was performed in Yaml, and standard web technologies such as HTML, CSS, and Javascript were used for the user-friendly presentation of the information (e.g., layout, design, and navigation). The main steps in the development of the Swiss eFFQ were the conceptualization of the user interface and the programming of the automatic calculation of the daily dietary intake data (see Section 2.1.5). The food composition database and the food mapping information were first integrated into the backend of the CMS. Then, for each food item, information about the question wording, the corresponding food mapping, the standard portion size, the available frequency options, the food photo(s) to be graphically displayed, and conditions on the presentation and characteristics of the question (e.g., required field and conditional visibility) were programmed into the Swiss eFFQ. The web platform allows for the automated processing and release of FFQ research data that are stored on a secure server.

#### 2.1.5. Calculation of Daily Dietary Intakes

**Figure 2:**
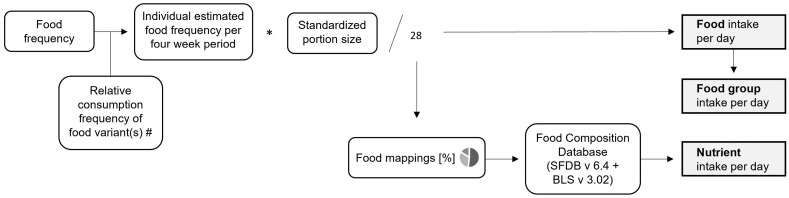
Overview of the steps for the automated calculation of daily dietary intakes in the Swiss eFFQ. # Food items with additional questions about the food variant commonly consumed (see Section 2.1.3): For type 1 questions, the relative frequency of consumption of variant A vs. variant B is calculated (never: 0%, rarely: 25%, sometimes: 50%, often: 75%, and always: 100%); and for type 2 questions, all selected food variants are weighted equally (e.g., if 3 food variants are selected, each one is calculated with 33.3% of the food item frequency). BLS: German food composition database (*Bundeslebensmittelschlüssel*); SFDB: Swiss food composition database.

The Swiss eFFQ web platform is programmed to automatically calculate daily dietary intakes by a direct link to a food composition database. Daily dietary intakes are calculated for foods, food categories, and nutrients. The details of the calculation are described below, and an overview of the main steps is shown in Figure 2.

In the first step, the frequency of consumption per four-week period is calculated for each food item, taking into account information on the food variants commonly consumed, if applicable. Some foods and beverages have a correction question included in the Swiss eFFQ; for these, the reported frequency of the food category is used as the correction factor for the reported consumption frequency of the food item. For these items, corrected intakes are calculated in addition to the crude intakes.

For each food item, the estimated amount consumed per day is then calculated by multiplying the individual frequency of consumption (per four-week period) by the standard portion size and dividing by 28. In addition, the total intake for each food group (e.g., sum of all vegetables) is calculated.

To estimate daily nutrient intakes, a food composition database was set up by combining the Swiss (version 6.4, SFDB [30]) and the German (version 3.02, BLS: *Bundeslebensmittelschlüssel* [31]) databases. More than 75% of the food items used in the calculation of the Swiss eFFQ were taken from the SFDB. To calculate weighted nutrient profiles for each food item, population-based food mapping percentages are used to estimate nutrient intakes. Daily nutrient intakes are estimated by (1) calculating the daily sub-food intakes (g/day) for each food item based on the food map percentages; (2) determining the corresponding nutrient intake by linking to the food composition database; and (3) calculating the total daily nutrient intake as the sum of nutrient intakes from all sub-foods.

To check the accuracy of daily dietary intakes calculated automatically by the Swiss eFFQ, the nutritional values were calculated separately using R software and compared with those calculated automatically. This internal validation process involved several steps, including comparisons of (1) crude and corrected frequencies of food items; (2) crude and corrected daily intakes of food items and food categories; and (3) crude and corrected daily nutrient intakes.

### 2.2. Pretesting and Translation of the Swiss eFFQ

The Swiss eFFQ was designed for all three linguistic regions of Switzerland, but so far has only been translated and is available in two languages (German and French). The development process of the multilingual Swiss eFFQ included phases of pilot testing, translation, and adaptation, which are described in more detail below (see Figure 1).

First, the German version of the Swiss eFFQ platform was developed and pilot tested in a convenience sample of Swiss adults. The aim of the pilot study was to assess the overall usability of the newly developed Swiss eFFQ (Ge v 1.0) in an online survey and to identify aspects for its improvement. Feedback on food items reported as missing and general comments on the Swiss eFFQ (assessed via open-ended text fields) were collected and used to improve the Swiss eFFQ (Ge v 2.0). Next, all content visible to the respondent (e.g., instructions, wording of questions, and food item vocabulary) was translated into French, checked for accuracy by two bilingual nutrition experts, and the main body (standard formulations of text elements) was back-translated into German. For the translation of the food items and food-related vocabulary, metadata from *menuCH* containing food vocabulary in the three national languages were used to improve the accuracy of the translations.

After comparing and proofreading the (back) translated texts, the adapted German Swiss eFFQ (v 3.0) was used to develop the first web-based version of the French Swiss eFFQ (v 1.0). Next, the usability of the French Swiss eFFQ was evaluated in a diverse group of people, including researchers, lay people, and nutrition experts. The feedback received was summarized, prioritized, and used to further optimize and finalize the web-based versions of both the German (v 4.0) and the French (v 2.0) Swiss eFFQ.

## 3. Results

### 3.1. Swiss eFFQ Development Phase

Baseline characteristics of the 2085 study participants whose 24 h-DR food consumption data were used to develop the Swiss eFFQ are shown in Table 1. The mean age (SD) of the study population was 46.9 (15.8) years, distributed to age groups as follows: 18–29 years (19.5%); 30–44 years (26.1%); 45–59 years (30.3%); and 60–75 years (24.0%). Of the total population, 54.6% was female, and the mean (SD) body mass index (BMI) was 26.0 (4.0) kg/m^2^ and 24.1 (4.7) kg/m^2^ for males and females, respectively. The linguistic region distribution of the participants was 65.2% German-, 24.5% French-, and 10.4% Italian-speaking region. Further details on the design and the main outcomes of the *menuCH* study are published elsewhere [19,20].

**Figure 3:**
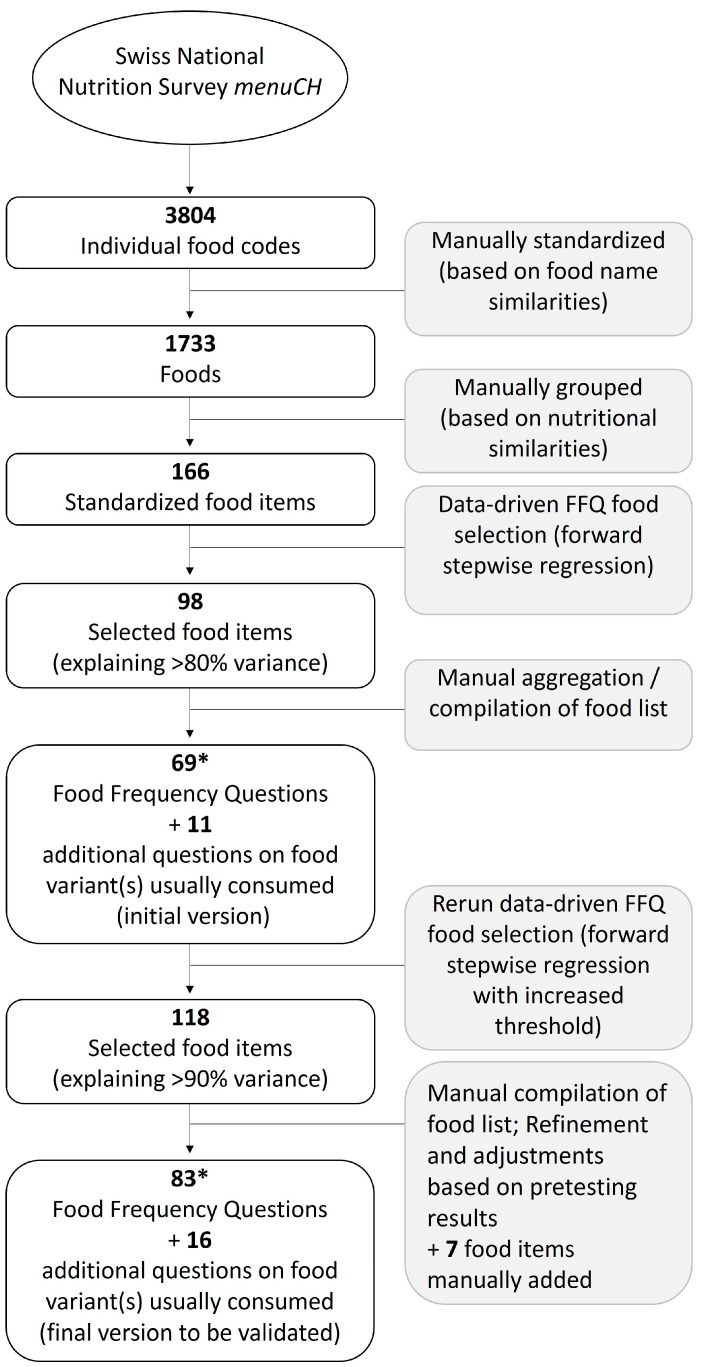
Flowchart of the Swiss eFFQ food item selection process. * The initial version of the Swiss eFFQ (Ge v 1.0) included 69 food frequency questions and only those foods that accounted for >80% of the variance in intake. The decision to increase the variance threshold to >90% led to a total food item number of 83.

Food consumption data used to develop the Swiss eFFQ contained a total number of 3804 food, dish, and beverage codes. Prior to analysis, these were manually standardized into 1733 foods and then further grouped based on nutritional similarities, resulting in 166 main food items that were used in the food item selection process (Figure 3).

**Figure 4:**
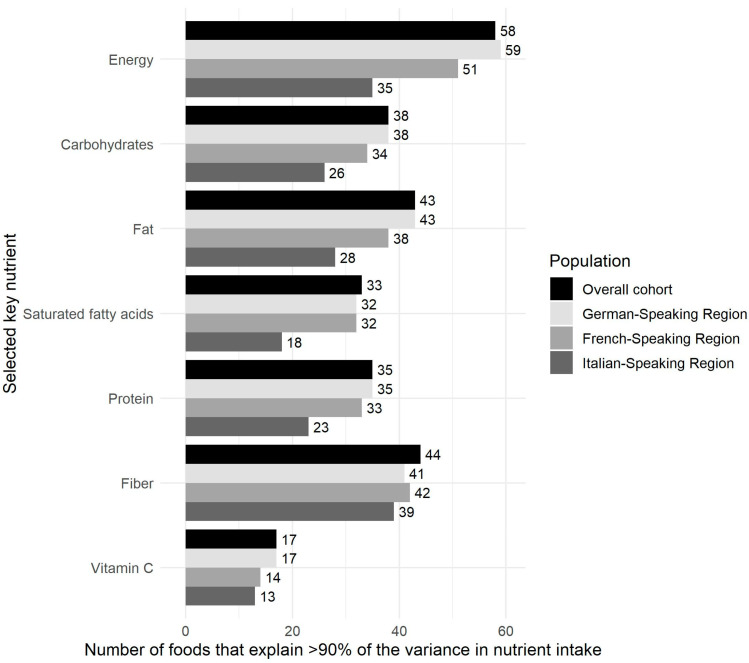
Overview of the number of selected food items explaining >90% of the variance in intake of energy or one of six nutrients in the overall cohort and in each of the linguistic regions.

From these, 102 food items were identified that explained >90% of the variance in intake of energy or one of the six nutrients of interest in the total cohort and 100, 96, and 82 food items in the German, French, and Italian linguistic regions, respectively, as summarized in Figure 4. While the largest number of food items was needed to explain the variance for energy, the lowest number of food items was required for vitamin C. After accounting for duplicates, a total of 118 different food items were identified by the data-driven approach, and 7 additional food items (e.g., water) were added to the Swiss eFFQ food list by considering expert opinions and feedback from usability testing. Finally, the food items were combined and rearranged into 83 food items that formed the final comprehensive Swiss eFFQ food list (Table S1).

Overall, the food items included in the final food list covered >90% of the absolute intakes of energy and six key nutrients in the intake of the cohort used to develop the Swiss eFFQ (Table 2). While the nutrients with the highest contribution to the absolute intake were protein (96.4%) and fat (95.9%), the nutrient with the lowest contribution was vitamin C (91.4%).

### 3.2. Pretesting and Translation Phase of the Swiss eFFQ

The pretesting of the initial German version of the Swiss eFFQ (Ge v 1.0) was conducted in a convenience sample of Swiss adults (*n* = 89) and revealed 23 general and 8 technical comments on the Swiss eFFQ. Reported difficulties included respondent confusion about certain aspects of the instructions, respondents’ need to be able to report missing foods, and technical problems related to long loading times. In addition, study participants reported approximately 40 different foods as missing.

During the translation process, several minor text differences were identified between the German and French texts, and corrections were made by the bilingual reviewers. A comparison of the original German main body (standard text elements in the Swiss eFFQ) with its back-translated version (Ge → Fr → Ge) revealed considerable differences in wording, terminology, and sentence structure in more than 90% of the sentences; however, no significant differences in content were found.

During the pretesting of the French version of the Swiss eFFQ (*n* = 18), most of the comments made were related to spelling or wording aspects (57 comments). In addition, a total of 11 technical, 14 general, and 24 food-related comments were reported, and their relevance for implementation was assessed by the researchers. All aspects rated as highly relevant were implemented (46.7%), and of the aspects rated as moderately relevant (19.4%), 15.0% were also implemented.

Overall, based on the results of the German and French pilot testing and the discrepancies identified during the translation process, several modifications were made to the Swiss eFFQ (Figure 1), resulting in an optimized and more user-friendly final version. For example, changes included the inclusion of additional food items (Figure 3), adjustments to explanations in the introductory section, changes in wording of the food frequency questions to improve clarity, and the correction of technical errors.

### 3.3. Characteristics and Layout of the Final Swiss eFFQ

The semiquantitative Swiss eFFQ was designed to assess dietary intakes over the previous four weeks with the primary aim of ranking Swiss individuals aged 18 to 75 years according to their self-reported consumption frequencies.

**Figure 5:**
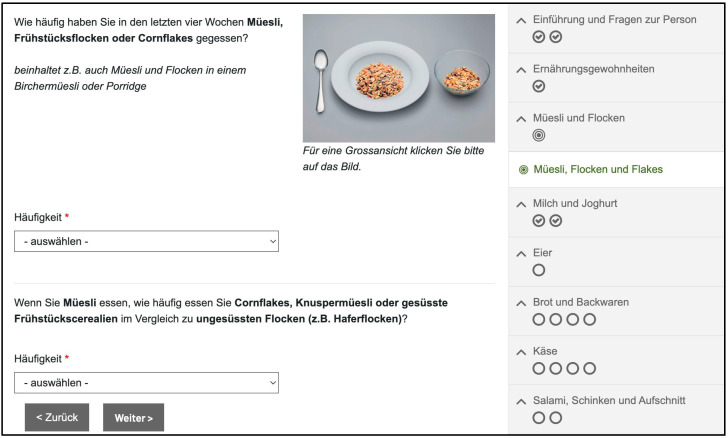
Screenshot of a food frequency question in the Swiss eFFQ, including an additional question about the food variant commonly consumed.

The introduction section of the Swiss eFFQ provides step-by-step instructions on how to complete the assessment tool, using an example food frequency question. In total, the Swiss eFFQ consists of 83 food frequency questions, of which 16 food items have additional food variant questions (e.g., yogurt variant(s)), leading to a total of 113 individual foods to be estimated. In addition to the main food item name, examples of similar foods belonging to the same food item group are given in the majority of the food frequency questions. When food examples were found to be unsuitable in one linguistic region, they were replaced with food equivalents with a similar nutrient composition from the other linguistic region. Figure 5 shows an example of a food frequency question included in the Swiss eFFQ.

**Figure 6:**
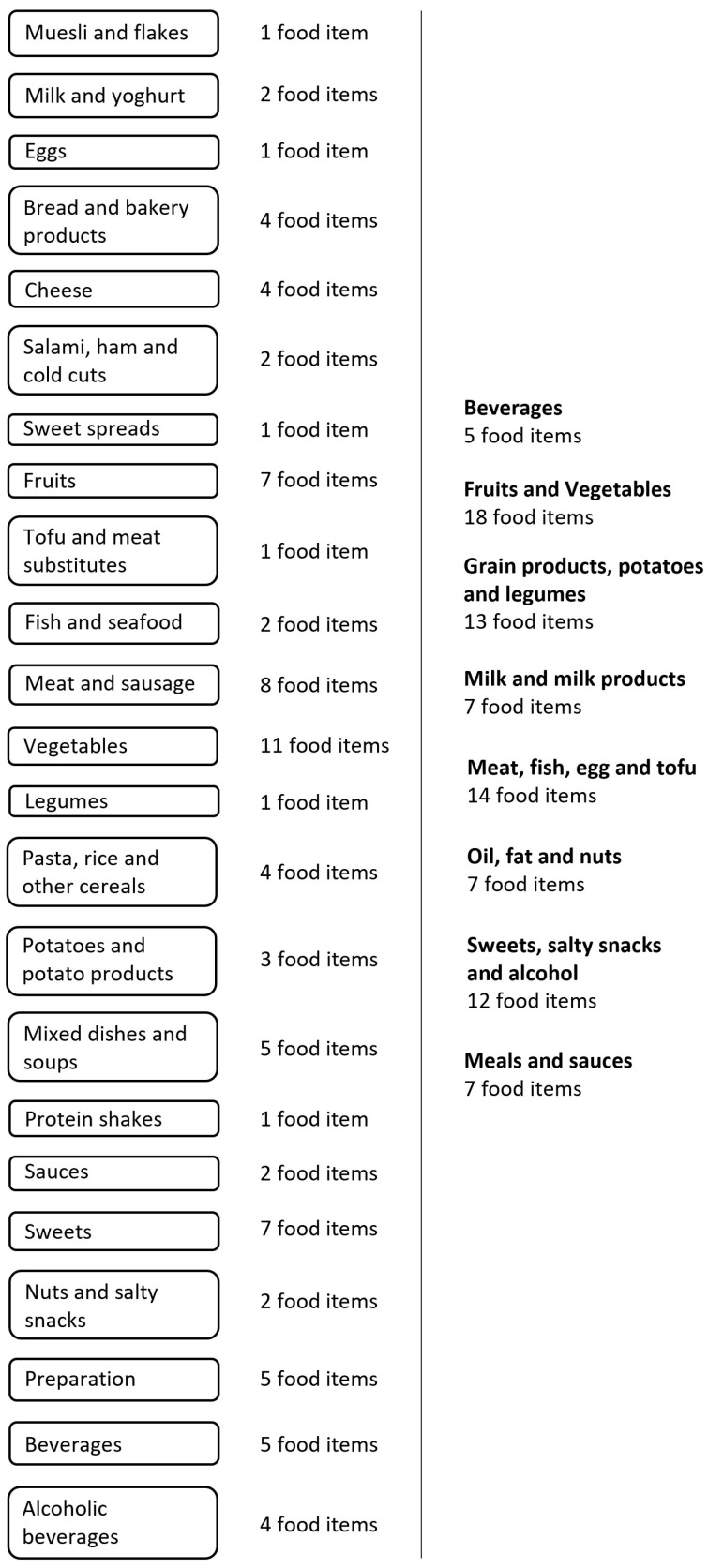
Overview of the order and number of food items per food group (**left**) and per Swiss food pyramid category (**right**) in the Swiss eFFQ [34].

The order of food items in the Swiss eFFQ follows a meal-based sequence, beginning with foods commonly eaten at breakfast. All food frequency response fields are mandatory, so that each question must be answered before proceeding to the next one. An overview of the final order and number of food items per food category is given in Figure 6. Open-ended text fields were included at the end of the Swiss eFFQ to allow respondents to enter the names and frequencies of foods that were usually consumed but were not explicitly queried in the Swiss eFFQ.

A number of different features were implemented into the Swiss eFFQ to improve usability from both the respondent and the researcher perspectives. Table 3 provides a comprehensive summary of the key features implemented into the Swiss eFFQ. The automated processing of FFQ dietary data provides the following output for the researcher: food intake (g/day, *n* = 113), food group intake (g/day, *n* = 21), and energy and nutrient intakes (kcal, kJ, g, mg, ug/day, *n* = 40) for each completed FFQ.

From the respondent’s perspective, the inclusion of aids such as food images, a navigation bar showing the survey progress, and built-in skip patterns (e.g., skipping questions related to animal foods for individuals following a vegetarian or vegan diet) are intended to improve the visual appeal and usability of the Swiss eFFQ while minimizing respondent burden.

## 4. Discussion

This paper describes the iterative process of the newly developed Swiss eFFQ optimized for assessing dietary intake in the culturally diverse adult population of Switzerland. Using a data-driven approach with the aim of ensuring good coverage of foods for all linguistic regions, nationally representative dietary intake data from 24 h-DRs were used to identify, both overall and by linguistic region, the foods that effectively capture >90% of the variance in consumption of energy and selected nutrients (carbohydrates, protein, fat, fiber, vitamin C, and saturated fatty acids). To improve the Swiss eFFQ based on the results of usability testing and expert feedback, the multi-stage development process included iterative phases of refining the food list and implementing wording, formatting, and technical changes to the Swiss eFFQ (see Figure 1). In total, the final Swiss eFFQ consists of 83 food frequency questions, 16 of which are complemented by additional questions about commonly consumed food variants. Based on the reported frequencies, standard portion sizes, and linked nutrient information from the Swiss and German food composition databases, the Swiss eFFQ web application allows the automated real-time calculation of daily dietary intakes at the food, food group, and nutrient level (see Figure 2).

The valid assessment of dietary intake is largely dependent on the ability of respondents to accurately report their food and beverage consumption and is one of the major challenges in nutritional epidemiology [35,36]. The use of new technologies in dietary assessment is considered to have the potential to facilitate respondent use while improving overall data quality [37]. To exploit this potential in the Swiss eFFQ, we implemented the automatic calculation of daily dietary intakes by linkage to the food composition database and used aids such as food photos and complex skip patterns and included required fields to maximize data completeness while improving overall usability (see Table 3).

However, respondent burden is not only influenced by the usability (e.g., intuitive use) of the tool and language style of the questions but also by the number of questions to be answered [38]. Following the recommendations of Willet et al. [39], who suggest less than 130 food items to minimize respondent burden, our comprehensive food list consisted of 83 food frequency questions (see Figure 3). Compared to other recently developed FFQs tailored for culturally diverse populations, where the number of food items ranged from 126 to ~200 [40,41,42], our Swiss eFFQ includes a relatively low number of foods. This is largely attributed to the data-driven strategy used to define the food item questions, i.e., the extent to which foods were combined into food items played a key role in limiting the length and duration of the final Swiss eFFQ. To create a medium-length FFQ, we focused on asking about the frequency of food intake and collecting data on the relative frequency of certain commonly consumed food variants. This approach allowed us to reduce the total number of food frequency questions while still obtaining intake data for 113 individual foods. Nevertheless, at the level of nutrient intake, the amount of variance explained by the food items included in the Swiss eFFQ is comparable to that reported in other studies that have used a data-driven approach to identify food items to include in a FFQ [40,42].

While some FFQs assess individual portion sizes with the aim of collecting precise information on the amount of food consumed [43,44,45], the Swiss eFFQ, in line with other recently developed FFQs [46,47], uses standard population-based portion sizes to estimate daily dietary intakes. Although accounting for individual portion sizes has been shown to add some precision, asking participants to self-report portion sizes has been observed to increase person-specific bias with only a limited improvement in capturing the overall variance in intake [48,49]. This finding, combined with our aim to minimize burden and cognitive challenges for respondents, justified the decision to use population-based standard portion sizes to estimate daily dietary intakes in the Swiss eFFQ.

To the best of our knowledge, we are the first to develop a web-based FFQ specifically designed to assess dietary intakes of the entire culturally diverse Swiss adult population. Although all three linguistic regions of Switzerland were considered in the food item selection process (see Figure 4), a limitation of the Swiss eFFQ is that it has only been translated, pilot tested, and is currently available in two of the national languages: German and French. However, given the intended use of the Swiss eFFQ in future nationwide Swiss studies, with due consideration of the Italian-speaking region in its design, the next step will be its translation into Italian, following the methodology outlined for the development of the French version (see Section 2.2).

In line with the observation of Pestoni et al. and Chatelan et al. that dietary habits differ in the linguistic regions of Switzerland [19,20], we identified a few food items unique to each linguistic region. However, most of the items were relevant in more than one linguistic region, suggesting that the differences in food consumption may be related to a small number of culturally specific foods or differences in the total quantities consumed rather than a completely different food consumption profile across linguistic regions. While some FFQs tailored to multi-ethnic groups use multiple versions of one FFQ with different food lists [41], due to the large overlap in identified foods and the need for a single tool to collect dietary intake data in Switzerland, we developed one FFQ with a comprehensive food list and included linguistic-region-specific food equivalents for example foods whenever appropriate (see Figure 6 and Table S1).

An important strength of the newly developed Swiss eFFQ is its ability to provide flexible, rapid, and cost- and time-efficient data collection, thus allowing for a low-effort yet efficient assessment of dietary intake in the context of nutritional epidemiologic studies (see Table 3). In this context, a major advantage of the Swiss eFFQ is its integrated, automated dietary data processing, eliminating the need for statistical and nutritional expertise and reducing the potential for data processing errors. The approach allows for a more detailed consideration of consumed food variants by taking into account relative frequencies, while maintaining a standardized and accurate calculation process that has undergone extensive internal validation. However, it is important to emphasize that no dietary assessment method can accurately measure an individual’s diet without error [5]. The data collected by FFQs are ultimately dependent on the cognitive ability of respondents and are therefore susceptible to biases, such as the under- or over-reporting of certain foods [50]. In addition, systematic errors may be introduced by the structure and assumptions of FFQs, such as standardized portion sizes and a limited range of foods included [50]. These limitations must be carefully considered when interpreting the data collected by the Swiss eFFQ. In addition, interpretations of absolute intakes are limited because the Swiss eFFQ was developed with the primary aim of ranking individuals according to their intake rather than estimating accurate daily intakes.

The Swiss eFFQ was developed using 24 h-DR data from a representative national sample of 2085 participants, capturing seasonal variations over a full year [20,25]. However, a limitation is that these data were collected in 2014–2015, and therefore do not cover recent trends in food consumption and dietary habits of the Swiss population. This was partially addressed in the development process by manually adding foods, such as plant-based milk alternatives, based on expert knowledge or feedback from study participants during the pilot testing of the Swiss eFFQ. Another limitation of the 24 h-DR data is that for saturated fatty acids, fiber, and vitamin C, the food composition database contained missing values for approximately 0.01%, 0.2%, and 21.2% of the food codes, respectively. Although nutrient values for these foods were imputed according to a procedure used in previous research projects using these data, the amount of missing data for vitamin C might have affected the observed results [19].

Lastly, a limitation that generally applies to the development of all FFQs is the potential for bias during the development process. Although we aimed for objectivity throughout the entire development process and by making decisions based on evidence or guidelines provided in the literature [5,8,51], certain development steps such as food standardization or decisions about which food variants to include might have been influenced by the researcher’s judgment.

In the present paper, we presented the iterative development process, the overall structure, and the main features of a newly developed multilingual 83-item web-based FFQ optimized to assess dietary intakes of adults living in Switzerland. Once validated, the Swiss eFFQ will enable the rapid, low-cost, real time, semi-automatic collection of data on habitual diet in the context of nutritional and epidemiological studies that can serve as a basis for dietary recommendations and intervention strategies at the individual and at the population level.

## Figures and Tables

**Table 1:** Baseline characteristics of study participants in the *menuCH* study whose food consumption data were used to develop the Swiss eFFQ ^a,b^.

	Overall	German- Speaking Region ^f^	French- Speaking Region ^f^	Italian- Speaking Region ^f^
Number of participants (n)	2085	1359	510	216
Sex Female	1139 (54.6)	718 (52.8)	298 (58.4)	123 (56.9)
Age (years) ^c^	46.9 ± 15.8	47.1 ± 16.0	46.4 ± 15.2	46.4 ± 16.0
Age groups (years) ^c^				
18–29	407 (19.5)	264 (19.4)	101 (19.8)	42 (19.4)
30–44	545 (26.1)	352 (25.9)	132 (25.9)	61 (28.2)
45–59	632 (30.3)	400 (29.4)	170 (33.3)	62 (28.7)
60–75	501 (24.0)	343 (25.2)	107 (21.0)	51 (23.6)
BMI (kg/m^2^) ^d^	25.0 ± 4.5	25.0 ± 4.4	24.8 ± 4.4	25.2 ± 5.0
BMI categories (kg/m^2^) ^d^				
Underweight	50 (2.4)	32 (2.4)	12 (2.4)	6 (2.8)
Normal weight	1116 (53.5)	721 (53.1)	282 (55.3)	113 (52.3)
Overweight	630 (30.2)	424 (31.2)	145 (28.4)	61 (28.2)
Obese	262 (12.6)	167 (12.3)	63 (12.4)	32 (14.8)
Nationality				
Swiss only	1506 (72.2)	1050 (77.3)	314 (61.6)	142 (65.7)
Swiss binational	303 (14.5)	165 (12.1)	110 (21.6)	28 (13.0)
Non-Swiss	276 (13.2)	144 (10.6)	86 (16.9)	46 (21.3)
Education, highest degree				
Primary/no degree	89 (4.3)	46 (3.4)	25 (4.9)	18 (8.3)
Secondary	980 (47.0)	624 (45.9)	245 (48.0)	111 (51.4)
Tertiary	1012 (48.5)	687 (50.6)	238 (46.7)	87 (40.3)
Smoking status				
Never	920 (44.1)	612 (45.0)	219 (42.9)	89 (41.2)
Former	692 (33.2)	434 (31.9)	183 (35.9)	75 (34.7)
Current	468 (22.4)	310 (22.8)	106 (20.8)	52 (24.1)
Self-reported physical activity ^e^				
Low	214 (10.3)	153 (11.3)	36 (7.1)	25 (11.6)
Moderate	499 (23.9)	316 (23.3)	128 (25.1)	55 (25.5)
High	837 (40.1)	549 (40.4)	210 (41.2)	78 (36.1)
Self-reported health				
Good or very good	1806 (86.6)	1214 (89.3)	442 (86.7)	150 (69.4)
Very bad to medium	274 (13.1)	142 (10.4)	66 (12.9)	66 (30.6)

^a^ Values are means ± SD for continuous variables and counts (% of answers) for categorical variables unless otherwise indicated. ^b^ Number of individuals with missing data for body mass index (BMI) *n* = 27; education *n* = 4; smoking status *n* = 5; physical activity *n* = 535; and health status *n* = 5. ^c^ Age is self-reported age at baseline. ^d^ BMI was calculated based on measured weight and height; when measurements were not feasible, self-reported information was used. Self-reported pre-pregnancy weight was used for pregnant/lactating women. ^e^ Physical activity level was assessed using the International Physical Activity Questionnaire (IPAQ) [32,33]. ^f^ German-speaking region (Cantons of Aargau, Basel-Land, Basel-Stadt, Bern, Lucerne, St. Gallen, and Zurich); French-speaking region (Cantons of Geneva, Jura, Neuchatel, and Vaud); Italian-speaking region (Canton of Ticino).

**Table 2:** A posteriori analyses on percent contribution of the food items included in the Swiss eFFQ to the total intake of energy, six nutrients, and the total amount of food consumed in the overall cohort and by linguistic region.

	Contribution (%) to Absolute Intake of Energy, Nutrients, and the Total Amount of Food Consumed			
Nutrient	Overall	German- Speaking Region ^b^	French- Speaking Region ^b^	Italian- Speaking Region ^b^
Energy	95.8	95.6	95.9	96.6
Carbohydrates	95.5	95.3	95.6	96.2
Fat	95.9	95.6	96.3	97.2
Saturated fatty acids	95.5	95.2	96.2	96.8
Protein	96.4	96.2	96.5	97.4
Fiber	93.7	93.8	92.8	95.2
Vitamin C	91.4	91.6	89.8	94.1
Total amount of food consumed ^a^	93.7	93.4	93.9	95.2

^a^ Contribution to the total amount of food consumed by the population (g) was calculated after excluding non-alcoholic and alcoholic beverages. ^b^ German-speaking region (Cantons of Aargau, Basel-Land, Basel-Stadt, Bern, Lucerne, St. Gallen, and Zurich); French-speaking region (Cantons of Geneva, Jura, Neuchatel, and Vaud); Italian-speaking region (canton of Ticino).

**Table 3:** Overview of key features included in the Swiss eFFQ.

Respondent Area	Researcher Area
- The eFFQ can be accessed through a generic or personalized URL link.	- Log in to the backend of the eFFQ using administrator-provided credentials. - Generate generic or personalized URL links for respondents to access the eFFQ.
- Detailed instructions on how to answer the eFFQ are displayed on the start screen. - “Help” button with instructions accessible throughout the eFFQ.	- The eFFQ can be customized to a limited extent to meet individual needs, such as modifying the instruction text or including/removing non-food-related questions (e.g., participant ID or demographics).
- A total of 83 food frequency questions, capturing consumption frequency over the previous four weeks using ten frequency categories, shown by drop-down menu.	- Cost- and time-efficient dietary data collection in large study groups. - Access real-time dietary intake data, automatically processed and linked to the food composition database, accounting for relative food frequencies of commonly consumed food variants.
- Examples of foods are given for most foot-item questions.	
- For 16 food frequency questions, respondents are asked to provide additional details on food variants commonly consumed.	
- Food images showing the standard portion size are displayed next to each food frequency question. Option to enlarge the images and view additional images of other example foods of the food item.	
- Questions on dietary habits (e.g., vegan, vegetarian, lactose, and gluten).	- Data collection on the following: Daily food, food group, and nutrient intakes, including both “crude” (raw data) and “corrected” values (correction questions); Dietary habits Supplement use; Sustainability behavior; Completion time of the eFFQ.
- Questions on supplement use.	
- Questions on sustainability behavior.	
- Correction questions on the frequency of intake of 10 foods or food categories that are known to be prone to over- or under-reporting (e.g., vegetables) or that are consumed in high frequencies (e.g., water).	
- Possibility to report food items consumed that were not previously queried in the eFFQ (using an open-ended text field + food frequency drop-down).	- Open-text field answers of respondents on missing foods enable the improvement of the eFFQ by implementing these suggestions or considering the consumption of these foods during data analysis.
- Complex skip pattern allows respondents to skip non applicable questions (e.g., meat/dairy products for respondents who have indicated following a vegan/vegetarian diet).	- Conduct study participant management and monitor data collection progress.
- Navigation panel shows progress and provides an overview of other food item questions asked.	
- Throughout the eFFQ, an error message prompts when a required field is left unanswered.	- Implement required fields to control for incomplete data entries.
- On the final screen before submitting, the respondent is prompted to verify that all questions have been answered and that they do not want to make any further changes. - Upon completion of the eFFQ, the respondent receives a confirmation message indicating successful submission.	- Possibility to export dietary intake data in a common file format (csv) that is ready to be analyzed. - Open or close the instrument for data collection. - Customize confirmation message displayed to respondents upon successful submission.

## Data Availability

Metadata (e.g., questionnaires) of the *menuCH* survey are available in a publicly accessible data repository (*menuCH* Data Repository): https://menuch.iumsp.ch (accessed on 14 September 2020). Restrictions apply to the availability of the *menuCH* research data. The authors’ access to the data set entitled “National Nutrition Survey *menuCH* 2014–2015” was obtained with the permission of the Swiss Federal Food Safety and Veterinary Office (FSVO) for the purposes of this research project.

## Supplementary Materials

The following supporting information can be downloaded at: https://www.mdpi.com/article/10.3390/nu15204359/s1, Figure S1: Consumption frequency per month covered by the ten frequency options included in the Swiss eFFQ; Table S1: Overview of the 21 food groups and their corresponding food items included in the Swiss eFFQ.
