# Supplementary material for: Development of a Multilingual Web-Based Food Frequency Questionnaire for Adults in Switzerland
Source: Nutrients. 2023 Oct 13;15(20):4359. doi: 10.3390/nu15204359 (PMC10610353; doi:10.3390/nu15204359)
Supplement: Supplementary file 1 [file nutrients-15-04359-s001.zip › nutrients-2647304-supplementary.pdf]

Supplementary Material

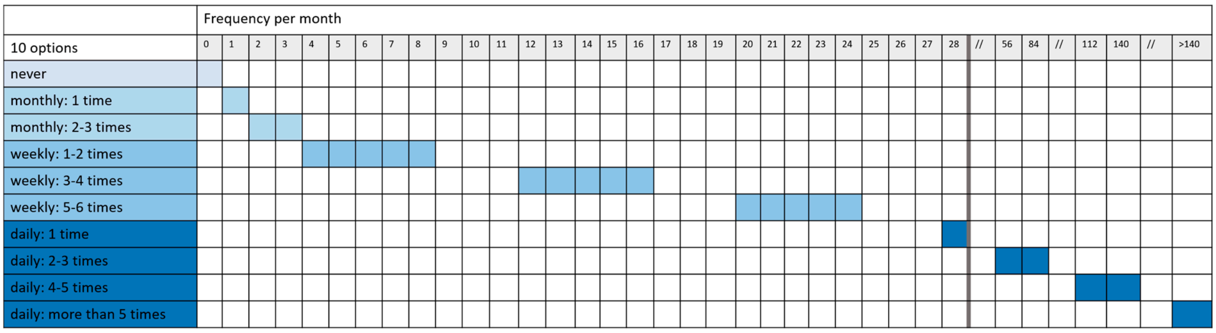

Figure S1. Consumption frequency per month covered by the ten frequency options included in the Swiss eFFQ.

Table S1. Overview of the 21 food groups and their corresponding food items included in the Swiss eFFQ.

Food group	Food items	Number of items
Non-alcoholic beverages	Water; Sugar sweetened beverages*; Coffee/coffee drinks*; Tea	4
Alcoholic beverages	Beer; Sparkling wine/champagne; Wine; Spirits/liqueurs	4
Fruits and fruit products	Grapes/berries; Apricot/peach/nectarine; Tangerine/orange; Southern fruits (banana/kiwi/fig/pineapple/mango); Apple/pear; Fruit compote; Dried fruits (dates/raisins/apricots); Fruit juice	8
Vegetables	Peas/green beans; Fennel/asparagus/chard/artichokes; Carrots/beetroot; Broccoli/cauliflower/cabbage vegetables; Spinach; Mushrooms; Tomato/cucumber/belled peppers; Zucchini/eggplant/pumpkin; Lettuce; Onions/leeks/garlic; Avocado; Warm sauces (roast/light/vegetable/tomato)*	12
Legumes	Legumes (lentils/chickpeas/kidney beans)	1
Bread and bread products	Bread/buns*; Yeast plait/croissant; Rusk/crispbread	3
Grains and grain products, rice	Breakfast cereals/cornflakes*; Corn/semolina/couscous/quinoa; Rice/rice dishes; Pasta/noodle dishes	4
Potatoes and potato products	French fries/croquettes; Rösti/potato gratin; Potatoes/gnocchi	3
Milk, dairy products and cheese	Milk/milk alternatives*; Yogurt/yogurt alternatives*; Hard/sliced/grated cheese; Raclette/fondue cheese; Mozzarella/cream cheese/curd cheese/cottage cheese; Soft cheese	6
Eggs	Eggs (boiled/scrambled/fried)	1
Vegetarian dairy product replacements and protein drinks	Milk/milk alternatives*; Yogurt/yogurt alternatives*; Protein shakes; Cream/cream substitutes*	4
Tofu and other vegetarian meat replacements	Tofu/plant-based meat alternatives*	1
Fish and seafood	Fish (salmon/tuna/cod/trout/perch)*; Seafood/shellfish	2
Meat, processed meat and sausage	Salami/country sausage; Ham/dried meat/cold cuts; Lamb/veal; Game/horse/goat meat; Chicken; Minced meat/ragout; Pork; Beef; Sausage/meat loaf; Offal	10
Nuts and seeds	Nuts/seeds	1
Fats, oils and cream	Vegetable oils*; Butter/margarine/fats; Cream/cream substitutes*	3
Salty snacks	Chips/salty snacks	1
Bakery products, cakes and pastry	Fruit tart; Cake/pie; Sweet pastries; Cookies	4
Sweets, sugar, dessert and ice cream	Sweet spreads (honey/jam/chocolate spread)*; Chocolate/pralines; Sweets; Ice cream/dessert; Artificial sweeteners; Sugar	6
Mixed dishes and soups	Soups/bouillons*; Pizza; Sandwiches/filled savory croissants; Quiche/tarte flambée; Kebab/burger	5
Savory sauces	Salad dressing; Cold sauces (mayonnaise/ketchup/pesto/mustard); Warm sauces (roast/light/vegetable/tomato)*	3

* Food frequency questions for these food items have an additional question about the food variant usually consumed; food variants belonging to different food groups are listed more than once (e.g., yogurt/yogurt alternatives are assigned to milk/dairy products and/or vegetarian dairy product replacements depending on the selected food variant).
